# The Effects of Household Food Security Discordance on Antisocial Behavior Among Latino Adolescents in the United States

**DOI:** 10.1155/nrp/3713065

**Published:** 2025-04-22

**Authors:** Fiorella L. Carlos Chavez, Keenan A. Pituch, Kasey E. Longley, Evan. P. Anderson, Daphne C. Hernandez

**Affiliations:** ^1^Edson College of Nursing and Health Innovation, Arizona State University, Phoenix, Arizona, USA; ^2^Florida Institute for Child Welfare, Florida State University, Tallahassee, Florida, USA; ^3^Department of Earth Systems and Society, Pima Community College West Campus, Tucson, Arizona, USA; ^4^Department of Research, Cizik School of Nursing, University of Texas Health Science Center at Houston, Houston, Texas, USA

**Keywords:** food insecurity, gender differences antisocial behavior, Hispanic youth, household food insecurity discordance, Latino adolescents

## Abstract

**Objectives:** First, to examine the effects of household food insecurity discordance status on adolescent antisocial behavior. Second, to determine if adolescents' gender moderates the association between household food insecurity discordance and adolescent antisocial behavior.

**Design:** Cross-sectional data of Latino parents and adolescents from the same household were collected in Tulsa, Oklahoma, between January 1, 2013, and January 1, 2014. Using a general linear model (GLM), we examined associations between household food insecurity discordance and adolescents' antisocial behavior.

**Sample:** The sample includes Latino parent and adolescent dyads (*N* = 69 dyads, 138 individuals), where parents were 89.9% women, Mage = 38.46, and adolescents were 43.5% girls, Mage = 14.3.

**Measurements:** Food security assessed using the 18-item US Food Security Survey for adults, the 9-item Self-Administered Food Security Module for children aged 12 and older for adolescents. Adolescents were asked to complete a modified 24-item version of the Problem Behavior Frequency Scale to assess antisocial behavior.

**Results:** Findings showed that adolescent food insecurity discordance was associated with greater antisocial behavior for girls. Parental food insecurity discordance was associated with greater antisocial behavior for boys.

**Conclusions:** Findings provide insight into the negative implications of household food insecurity discordance on Latino adolescents' antisocial behavior.

## 1. Background

In 2022, 17.3% of U.S. households with children were food insecure, with greater prevalence in Latino1 households with children (24.7% food insecure) [1]. Household food insecurity is associated with poor mental health among adolescents [2] and associated consequences, such as antisocial behavior [3–5]. Among Latino adolescents, prior research has found significant relations between household food insecurity and poor mental health [6, 7]. Adolescents limiting food and experiencing hunger without parents' knowledge have been described as “household food security discordance” [8]. This phenomenon may affect adolescent girls' psychological adjustment more than boys. Women more often experience food insecurity [9, 10], and Latino cultural values socialize women to sacrifice for family well-being [11, 12]. However, there is little information about the relation between food insecurity and antisocial behavior among Latino adolescents. The present research specifically studies the association between household food insecurity discordance and Latino adolescents' antisocial behaviors in order to begin to address this gap in the literature.

### 1.1. Food Insecurity Discordance in Latino Adolescents

There is evidence to suggest that financial hardship (i.e., poverty) is linked with antisocial behavior among adolescents [13, 14]. Therefore, it is possible that financial hardship not only worsens food insecurity among adolescents and their families [15, 16], particularly among Latino adolescents [7], but that food insecurity may, in turn, be associated with antisocial behaviors. Assessing these connections requires, first, an accurate assessment of household food security status. However, the typical strategy for measuring household food security makes a fundamental assumption, which is questionable in households with adolescents. That is, household food security is assessed by a single adult in the home [17], typically the mother when children are present [18]. The assessment strategy assumes the respondent is aware of all actions taken by all family members to stretch food supplies (e.g., eating less, skipping meals) as well as the family members' subsequent experiences of hunger.

The assumption is not without merit, recognizing that mothers frequently reduce their food consumption to ensure adequate food for children [19, 20]. However, there is also reason to expect that the measurement strategy may become more precarious as children in the household age into and through adolescence. For example, evidence suggests that children as young as 9 years old become cognitively, emotionally, and physically aware of food insecurity and that they may take some responsibility in managing household food resources [21, 22]. As such, it is possible that adolescents may be helping to insulate younger children in the household from food insecurity by deliberately cutting back on their own intake, thereby “protecting” more vulnerable younger children from hunger [23, 24]. Herein lies a potential mechanism for discordant appraisals of household food security, where adolescents mask their food insecurity not only for younger siblings, but for the sake of their parents and the household, too [21, 25].

This dynamic of household food insecurity discordance has received little attention for Latino families. Only three studies could be identified that involved exclusively Latino households and included both Latino parents and children in the assessment of household food security. Nalty et al. [26] collected data from mother–child dyads living in the *colonias* along the Texas–Mexico border. They reported modest kappa statistics (Κ ranged from 0.09 to 0.13) between mothers' and children's responses to standard food security questions (e.g., reduce portion size, skip meals, feelings of hunger) suggesting “slight” agreement. They also estimated that 80% of mothers reported household food insecurity, while 64% of the children reported experiencing food insecurity. On the other hand, in a study of predominantly mother–adolescent dyads in an urban area in Oklahoma, 53% of households characterized as “high food security” by the parent were classified as having poorer food security based on information provided by the adolescent [8]. Further, Carlos Chavez et al. [8] reported that poorer parent–child relationship quality—assessed in terms of frequency of both parent- and child-reported conflict—increased the likelihood of adolescents reporting food insecurity when parental reports suggested food security. Lastly, a study in South Carolina among Latino households pointed out that children did not tell their parents/caregivers about their cognitive awareness of food insecurity in the household and that their parents were only aware of 40.4% of instances of adolescents' food insecurity experiences [22]. In addition, Latino adolescents in this study took responsibility for food insecurity by engaging in adult strategies (e.g., hide food for family members), generating their own strategies (e.g., ration food portions and buy cheaper products), or coming up with resources (e.g., sell their toys) for the maintenance of food supply [22].

### 1.2. Antisocial Behaviors Dynamics Among Latino Adolescents

Antisocial behavior has been argued to originate in childhood, and it is characterized by behavior problems and poor self-regulation across several domains of functioning (e.g., physiological, cognitive) [27]. Antisocial behaviors among Latino adolescents in low-income households have been documented [28], and prior research has found that Latino adolescent boys are at higher risk of antisocial behaviors than Latino adolescent girls [29, 30]. However, there is evidence to suggest that Latina adolescent girls who experienced mildly to moderately depressive symptoms may be at risk of engaging in antisocial behavior [31]. Thus, given that depressive symptoms increase with age for Latina adolescent girls [32], it is possible that they may begin to manifest antisocial behaviors if their depressive symptoms do not receive needed treatment [31]. While prior work has shown a significant association between food insecurity and antisocial behavior among adolescents [33, 34], little is known about this association among Latino adolescents, particularly in the case of household food insecurity discordance.

### 1.3. Theoretical and Empirical Foundations

Stein's et al.'s model of psychological adjustment among minority youth guides our study. Essential to the model is the concept of intersectionality or the view that individual experiences are assigned based on overlapping or “intersecting” expectations summarized by demographic identities like “race,” “ethnicity,” and “gender” [35]. Stein and colleagues used this idea to argue that minority status, rurality, and growing new immigrant destinations for Latino families shape the day-to-day experiences of Latino adolescents, their social position, and respective psychological adjustment. Furthermore, Stein et al. [36] argues that social positionings group adolescents into stratified hierarchies with unequal worth and importance, thus perpetuating a belief system that dictates the value of individuals and communities based on this social position [37].

In this study, we work with the social position, economic segregation, and cultural expectation elements of Stein and colleagues' model within Latino households. First, we leverage the idea of intersectionality and argue that household food insecurity creates a context for human development that is distinctive for Latino adolescents. The distinction derives from Latino cultural norms and beliefs such as familism (*familismo*) and respect (*respeto*) that encourages familial priority and reciprocity [38]. Additionally, simpatía highlights the expectation of maintaining family harmony and pleasant interactions in relationships [39, 40]. Taken together, these cultural values, beliefs, norms, and expectations may provide Latino adolescents a specific role and range of behavior with which they are expected to comply within the family [41].

Second, we leverage intersectionality by focusing on potential differences between adolescent boys' and adolescent girls' experiences in food insecurity discordance, given the delineated gender role expectations in Latino households [42, 43]. For example, gender norms of *marianismo* idealize Latino women who are virtuous, humble, self-sacrificing, and who can withstand suffering for the family's well-being [44, 45]. Thus, adolescent girls, in their effort to minimize discomfort within family relationships [46], may take on the responsibility to secretly “protect” their siblings and/or parents from food insecurity through personal reduction or abstention. This additional burden of self-sacrifice [12] may take a greater toll on their psychological adjustment compared to adolescent boys [47, 48].

Finally, we use Stein and colleagues' developmental model to suggest that household food insecurity discordance is a family phenomenon that interacts with economic background and residential segregation (e.g., living in nontraditional immigrant destinations) which limit the availability of food resources for Latino adolescents. Managing food resources in Latino households is a role traditionally reserved for the parent [49], usually mothers who shield the children in the household from experiencing food insecurity [19]. Cultural values may inhibit Latino adolescents from sharing experiences of food insecurity with their parents. Familism (i.e., *familismo*), respect (i.e., *respeto*; obedience to authority figures [50]), and simpatía potentially lead adolescents to hide their personal efforts to minimize food insecurity. This helps parents to “save face” and retain authority, which would be challenged by, for example, not being able to meet household food needs, or not being seen as the home's “bread-winner.”

Thus, Latino adolescents who take on the responsibility to “manage” food resources without the knowledge of their parents risk household food insecurity that cannot be alleviated elsewhere, and risk future emotional distress and antisocial behavior. Particularly, strains resulting from growing life–family demands, socioeconomic stressors, and the consequences of potentially poor (i.e., risky) decision-making (e.g., cutting down on food intake, skipping meals) may create the potential for discordant reports of household food insecurity among Latino parents and adolescents, as well as the emotional burden associated with it. This potential has already been documented [8, 22], but its dimensions and consequences have not yet been satisfactorily explored.

## 2. The Present Study

While previous work made efforts to delineate household food concordance and discordance among Latino parents and their adolescents [8], it considered only a few scenarios of how concordance or discordance could be expressed, and did not consider intersections with antisocial behavior. In the present research, we more systematically define types of household food concordance and discordance. These are as follows: (A) *Household food security concordance* wherein both parents and adolescents report food security in the household; (B) *Household food insecurity concordance* wherein both parents and adolescents report food insecurity in the household; (C) *Adolescent food insecurity discordance*, when adolescents report food insecurity but parents do not; and (D) *Parental food insecurity discordance*, when parents report food insecurity but adolescents do not.

Guided by the modified integrative model of minority child development [36], the present study had two core aims with respective hypothesis.

• Aim 1: To examine the effects of household food insecurity discordance status on adolescent antisocial behavior.
• Hypothesis 1: Adolescent food insecurity discordance will be associated with adolescents' antisocial behavior.
• Hypothesis 2: Parental food insecurity discordance will be associated with adolescents' antisocial behavior.
• Hypothesis 3: Household food insecurity concordance will be associated with adolescents' antisocial behavior.
• Aim 2: To determine if adolescents' gender moderates the association between household food insecurity discordance and adolescents' antisocial behavior.
• Hypothesis 4: Compared to girls, antisocial behavior for boys will be more negatively affected by adolescent food insecurity discordance.

## 3. Methods

### 3.1. Sample

The data are from the Tulsa 100 Family Study, a project described in previous research [8, 51]. The focus of the Tulsa 100 Family Study was to examine the individual, familial, and environmental factors impacting adolescents' well-being in neighborhoods with high concentrations of poverty. The project recruited a total of (*N* = 92; parent-adolescent dyads for a total of 184 participants) through community-based strategies. All recruitment and data collection procedures were approved by a University Institutional Review Board. An NIH Certificate of Confidentiality was obtained to further protect participating families' personal information and confidentiality.

The current analyses were restricted to Latino parent–adolescent dyads (*n* = 69 dyads; 138 participants). Participating parents were women and men who self-identified as Latino were the parent or legal guardian of an age-eligible adolescent, agreed to participate in the study, and accepted to provide authorization for the minor to participate in the study. Stepparents and grandparents were eligible to participate in the study only if they were the primary caregivers of the focal adolescent. Participating adolescents were boys or girls who self-identified as Latino were currently attending a Tulsa Public School, and agreed to participate in the study.

### 3.2. Procedures

#### 3.2.1. Data Collection Design

Trained bilingual (Spanish–English) community-based interviewers (CBI) collected all data. Specifically, data from parents were collected with the support of CBI through interviewer-administered survey questionnaires while data from adolescents were collected using tablets where adolescents completed a self-administered survey questionnaire through Qualtrics. The data for the parent and her/his respective adolescent were collected simultaneously, but independently, at the participants' homes and in their preferred language (i.e., Spanish or English) between January 1, 2013 and January 1, 2014. Adolescents were interviewed separately and in their preferred language in order to reduce the perceived risk of having their parent overhear their responses and to reduce the likelihood that they would provide socially desirable answers to appease their parents.

#### 3.2.2. Measures

##### 3.2.2.1. Food Security

Household food security was assessed by parents using the 18-item US Food Security Survey (FSS) [52, 53]. Items from the parent household measure included questions about food running out, not having enough money to buy more food, being unable to afford balanced meals, relying on low-cost foods, feeling hungry, skipping meals, and eating less, among other items. A sample item included “The food that (I/We) bought just didn't last, and (I/We) didn't have money to get more.” Food security was constructed from parents' responses using publicly available instructions for households with at least 1 child [52]. The levels of food security (or the lack thereof) were delineated into two categories: Scores of 0–2 were labeled “food secure;” and scores of 3 through 18 were labeled “food insecure.”

Food security was assessed by adolescents using the 9-item Self-Administered Food Security Module for Children Ages 12 and older (CFSSM) [54]. Items from the children food security measure included questions about food running out, not having enough money to buy more food, eating cheap meals, not eating balanced meals, relying on low-cost foods, feeling hungry, and eating less, among other items. A sample item included “Did you have to eat less because your family didn't have enough money to buy food?” Food security status was constructed and coded from adolescents' responses. Following USDA standards [54], scores of 0–1 were labeled “food secure,” whereas scores of 2 through 9 were labeled “food insecure.”

##### 3.2.2.2. Food Insecurity Discordance

Food insecurity discordance was constructed by cross-classifying parents' and adolescents' reports of household food security as “food secure” and “food insecure” resulting in four distinct groups reflecting: (1) *household food security concordance* (parents and adolescents both reported household food security), (2) *household food insecurity concordance* (parents and adolescents both reported food insecurity), (3) *adolescent food insecurity discordance* (adolescents report food insecurity but parents report food security), and (4) *parental food insecurity discordance* (parents report food insecurity but adolescents report food security).

##### 3.2.2.3. Antisocial Behavior

To assess for adolescents' antisocial behavior, we used a modified version of the 26-item Problem Behavior Frequency Scale [55, 56]. A sample item included “How often in the past year did you participate in each behavior…Get into trouble at home?” We used a 5-point Likert scale ranging from (1 = never to 5 = very often) and averaged across items to obtain scale scores, where higher scores indicate greater antisocial behavior as reported by the adolescent (*α* = 0.84). For the present study, the scale scores range from 1 to 2.5. This scale has been previously used among adolescents [57, 58], including Latino adolescents [59, 60].

##### 3.2.2.4. Additional Covariates and Factors

Adolescents' self-reported age was used as a continuous variable, and adolescents' self-reported gender was coded as a binary variable (0 = girl, 1 = boy). Household income as reported by the parent was coded as a binary variable (0 = higher than $30,000 and 1 = less than $29,999). Previous studies using this dataset have used $30,000 as the delineation standard for household income [8].

### 3.3. Analytic Strategy

Study hypotheses were tested by estimating a general linear model (GLM) for the outcome of adolescent antisocial behaviors. This model included the factors: (1) food security, with four groups (household food security concordance, household food insecurity concordance, adolescent food insecurity discordance, parental food insecurity discordance), (2) gender (i.e., boy, girl), and (3) their interaction. The model also included covariates adolescent age and household income. Given a significant interaction, we assessed simple effects and plotted model-based predicted values for each of the food groups and gender, holding covariates constant at their mean.

## 4. Results

### 4.1. Descriptive Statistics

Table 1 shows that Latino adolescents (*n* = 69) were majority boys (56.5%), ages 11–19 years, *M*age 14.26 years (SD = 2.01), and about 40.6% were at or above 10th grade. Latino parents were primarily women (90%), of ages 26–65 years, Mage 38.46 (SD = 6.50), and 80% were the biological mothers. A great majority of the parents interviewed were married or living as married (88%). Over half of the parents did not graduate from high school (67%). Almost a third of the parents interviewed (32%) held more than one job, and close to 67% of the families earned less than $30,000 per year. Antisocial behavior ranged from 1 to 2.5 (mean = 1.38; SD = 0.35).

**Figure 1:**
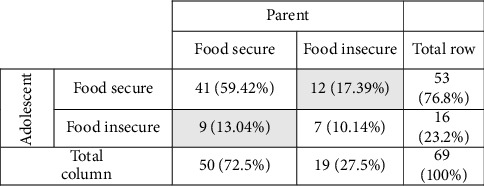
Household food security concordance, and household food insecurity concordance and discordance among Latino parent–adolescent dyads (*N* = 69 dyads; 138 participants). Note. Food insecurity discordance groups are in the shaded areas. Additionally, there is poor inter-rater agreement in the parent and adolescent's self-report of food security (*K* = 0.198, *p* = 0.098; [61]).

Based on the descriptive responses from parents, most households were classified as “food secure” (72.5%). Based on the responses from adolescents, most households were classified as “food secure” (76.8%). Cross-tabulations of parents' and adolescents' reports of food security status (Figure 1) indicate that 59.4% of dyads (i.e., parents and adolescents) reported household food security concordance, while 10.1% of dyads reported household food insecurity concordance. Moreover, 13.0% of dyads reported household food insecurity among adolescents but household food security among parents (i.e., *adolescent food insecurity discordance*), and 17.4% of dyads reported household food insecurity among parents but household food security among adolescents (i.e., *parental food insecurity discordance*). In addition, there was a poor inter-rater agreement in the parent and adolescent's self-report of food security (*K* = 0.198, *p* = 0.098) [61].

### 4.2. GLM Analysis

#### 4.2.1. Antisocial Behavior

**Figure 2:**
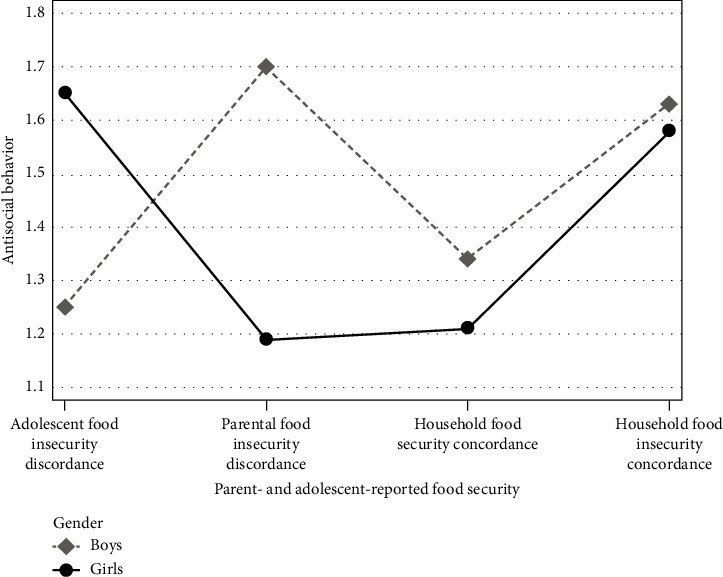
Antisocial behavior adjusted means by food security and adolescent gender (*N* = 69 parent–adolescent dyads; 138 participants).

The main effects of food security, *F*(3,59) = 2.47, *p* = 0.071, Δ*R*^2^ = 0.09, and gender, *F*(1,59) = 0.45, *p* = 0.505, Δ*R*^2^ = 0.006, were not significant. However, the interaction between these variables, as depicted in Figure 2, was significant, *F*(3,59) = 2.99, *p* = 0.038, Δ*R*^2^ = 0.11, indicating that the pattern of mean differences across the food groups differed by gender. When assessing food group differences by gender, Table 2 shows that girls' antisocial behavior is greater for the adolescent food insecurity discordance group (*M* = 1.65) and the household food insecurity concordance group (*M* = 1.58) compared to the household food security concordance group (*M* = 1.21), with the differences, respectively, being 0.44, *t* [59] = 2.46, *p* = 0.017, and 0.37, *t* [59] = 2.06, *p* = 0.044. In contrast, for boys, antisocial behavior was greater in the parental food insecurity discordance group (*M* = 1.70) compared to the adolescent food insecurity discordance group (*M* = 1.25) and the household food security concordance group (*M* = 1.34), with these differences, respectively, being 0.45, *t* [59] = 2.43, *p* = 0.018, and 0.35, *t* [59] = 2.54, *p* = 0.014. Comparisons between gender by food group indicated that only in the parental food insecurity discordance group do adolescent boys report greater antisocial behavior than girls (MD = 0.50, *t* [59] = 2.28, *p* = 0.026). Intergender comparisons within food groups were otherwise not significant. Covariates adolescent age (*b* = −0.02, *t* = −0.83, *p* = 0.408) and household income (*b* = −0.08, *t* = −0.86, *p* = 0.394) were not associated with antisocial behavior. Finally, although the main effect of food security was not significant, averaging across gender, antisocial behavior was greater for household food insecurity concordance than household food security concordance (Table 2), MD = 0.33, *t* [59] = 2.35, *p* = 0.022.

## 5. Discussion

Household food insecurity discordance in families is a poorly understood phenomenon, especially across low-income families from Latino backgrounds. The present study examined the associations between household food insecurity concordance and discordance on Latino adolescents' antisocial behavior and whether these associations vary based on adolescents' gender. Using both parent and adolescent's self-reports of household food security status, four groups of parent- and adolescent-reported household food security were created and examined, and compared with assessments of antisocial behavior and reported adolescent gender. Findings were partially consistent with our first three hypotheses (Table 2): Adolescent food insecurity discordance was associated with antisocial behavior (Hypothesis 1), but only for girls in comparison with household food security concordance. Parental food insecurity discordance was associated with antisocial behavior (Hypothesis 2), but only for boys in comparison with the food security concordance groups. Household food insecurity concordance was also associated with antisocial behavior (Hypothesis 3), but only in two circumstances: for girls and for both genders averaged together in comparison with household food security concordance. Data were not consistent with hypothesis four, as boys were found to have significantly greater antisocial behavior, but only for parental food insecurity discordance.

Although previous research has pointed out that household food insecurity is predictive of psychological distress for adolescents [62, 63], as well as of various forms of misconduct in adolescent boys [64], this is the first study among Latino adolescents to examine the association between discordance in household food security and antisocial behavior. Our findings make four contributions to the household food security literature, particularly the emerging literature on the consequences of household food insecurity discordance on antisocial behavior in Latino adolescents.

### 5.1. Latino Cultural Influences on Adolescent Development

First, the cultural context of Latino adolescent development could help explain discordant reports of household food insecurity and their effects on Latino adolescents' antisocial behavior. Prior research has shown adolescents as young as 11 years old are not only aware of family financial hardships but also worry about their family's ability to cover basic needs [65]. Additionally, children in food-insecure households often take “secret action” to managing household food resources [66], even experiencing hunger themselves [21, 67]. From the perspective of Latino cultural norms, the adolescents may have hidden their worry about their family's ability to cover basic needs such as food, to the point of experiencing food insecurity themselves without their parent's knowledge, out of a sense of family interdependence (*familismo*) and out of respect for their parents' roles and authority in the family. Thus, when Latino parents and their children experience food hardships, Latino adolescents may engage in an expression of care wherein they take some of “the burden” upon themselves without disclosing their actions to parents. This could create either a concordant food-insecure environment, in which both parent and child are aware of the food-availability stress (even if parent and child do not necessarily know that the other is aware of the stress), or a discordant food insecurity environment, where the adolescent is food insecure, but the parent is not.

In addition, parents generally attempt to shield their children and adolescents from food insecurity in the household [68], and among Latino households with children, parents not only give up their food so that their children can eat without noticing the food scarcity, but they also tend to believe that their kids do not understand what is happening and are therefore not affected by it [22]. Nevertheless, if parents are not successful in equally protecting their children (younger and older) from experiencing food insecurity, adolescents may feel like they are taking on an “adult-like” role of protecting the family by eating less for the sake of their younger siblings. Indeed, previous research has found that parents underestimate the extent to which their children worry about food scarcity and the type of behaviors in which they engage to conserve food resources [69].

Household food insecurity discordance can contribute to negative effects on adolescents' behavior, particularly since adolescence is a complex and vulnerable period of human development wherein the brain, behavioral, and cognitive systems are developing in response to situations confronting adolescents [70]. They are thus at heightened susceptibility to engage in risky and irresponsible behavior, and adolescents who are more susceptible to stress can also show more antisocial behaviors [71]. In particular, Latino adolescents are more likely to have poorer mental health than their age counterparts in other ethnic groups [72], and their stratified social position based on their family's socioeconomic status, race, ethnicity, and gender may affect their development [36]. These circumstances and their developmental stage may account for the higher levels of antisocial behavior seen in this study among Latino adolescents in some food-insecure concordant households and food-insecure discordant households.

### 5.2. Gender Differences in Antisocial Behavior Among Latino Adolescents

Second, differences in antisocial behavior between the four food-availability and concordance groups were statistically significant in this study only when the effect of gender was considered (excepting the gender averaged comparison of food insecurity concordance to food security concordance). Even then, different genders reacted with greater antisocial behavior to different food groups (Figure 2; Table 2). Given that gender socialization is more pronounced for Latino families compared to other U.S. ethnic groups [43], it is likely that Latino boys and girls experienced the effects of household food insecurity discordance differently. Specifically, the inability of Latino adolescent girls to disclose to their parents their experiences with food insecurity or the awareness of Latino adolescent boys to realize their parents may be shielding them from experiencing food insecurity has significant consequences for adolescents' antisocial behavior. Perhaps not surprisingly, adolescents who felt they were unable to openly communicate with their parents, as would be suggested in a food-security discordant household, experienced poor psychological adjustment [73].

### 5.3. Gender Roles and Socialization Among Latino Adolescents: Focus on Individual Genders

Third, compared to the concordant condition of adolescent and parent food security, food-insecure adolescent girls reported greater externalized antisocial behavior, regardless of whether their parent was food secure (discordant) or insecure (concordant). A robust body of research has documented that Latino adolescent girls may be at higher risk of developing poor psychological adjustment than Latino adolescent boys as a result of discrimination [74, 75], traumatic experiences (PTSD) [76], bicultural stress [39], and acculturative stress [77].

Previous research has also found that food insecurity was associated with externalizing behaviors among Latino girls [7]. In the context of household food insecurity discordance, adhering to traditional family values such as familism and *respecto* [41] in order to help the family maintain resource availability and reduce family worry may put adolescent girls at risk of high levels of antisocial behaviors. Latino girls may be taking on more of the caretaking roles in the household in order to keep family harmony and reduce family stress compared to boys [78]. Here lies the potential for Latino adolescent girls to engage in antisocial behavior, especially if they feel they have to abide by cultural traditions and values that may oppose their need for autonomy and independence as adolescents [79–82].

Fourth, although prior research has found that Latino adolescent boys are at higher risk of antisocial behaviors than Latino adolescent girls [29, 30], antisocial behavior was higher among boys compared to girls only in the discordant condition where adolescents were food secure but parents were insecure. Previous research has presented that parents may shield adolescent boys from food insecurity (e.g., hunger) before girls, who may be more in tune with their parents' anxiety over a lack of food than adolescent boys [83]. This alone can help explain why when girls reported adolescent food insecurity discordance and household food insecurity concordance, antisocial behavior was high, but this was not mirrored in boys. Despite being “protected” from experiencing immediate food insecurity, boys living in a food-insecure household may yet manifest antisocial behavior, perhaps sensing something is “off” but feeling powerless to know what it is or stop it. This finding is in alignment with prior research wherein adolescent boys in households where parents reported food insecurity experienced higher antisocial behaviors than adolescent girls [64], but to our knowledge, this is the first study exclusively focusing on Latino families showing that adolescent boys who report food security and live in food-insecure households experience higher antisocial behaviors than adolescent girls.

### 5.4. Limitations

This research should be considered in light of not only its strengths and implications, but also its limitations. First, we focused exclusively on Latino households located in the South-Central region of the United States. Therefore, these findings cannot be generalized to other Latino groups in the United States nor to other geographic regions. Second, the questionnaires regarding youth food security were administered only to one child in the household. As such, it is not possible to generalize that all children and/or adolescents in the household may have experienced food insecurity or that all children/adolescent's food security reports were different than their parents'. Third, we do not know whether the focal child was the oldest, youngest, or middle child in the family. Knowing the child's birth order may help us understand the adolescent positionality in “food management” in the household for the well-being and caregiving of their younger siblings. Fourth, it is possible that respondents' understanding of food insecurity differs from that of the USDA. Indeed, respondents were surveyed using a Spanish or English language questionnaire (based on their preference), and some meaning or interpretation of “household food security” based on food preferences and cultural factors may have been lost in translation [84, 85]. Fifth, data were cross-sectional and information about parents' and children's immigration status in the household was not collected. Knowing whether the family was experiencing a documentation status stressor could have helped explain why some Latino adolescents did not share their experiences with food insecurity or hunger with their parents, so as not to burden their parents with an “extra stressor” (though such a motivation would certainly be in line with the expectations of simpatía). Finally, it may be possible that Latinos, as a collectivistic group, may be more inclined to provide *socially desirable behaviors* than sharing the truth about their food intake which may help explain why parents were unaware of their adolescents' household food insecurity experiences.

### 5.5. Future Directions

The data for this paper were collected in 2013 and 2014; the associations between food-availability concordance and discordance with gender and antisocial behavior may not apply now exactly as they did, then. However, given the increasingly hostile legal and cultural environment toward Latinos, particularly immigrant and migrant Latinos, we would expect increases in food insecurity, discordance, and stress. Whether or not these circumstances manifest as greater antisocial behavior in adolescent boys or girls is more difficult to say, and would be a cogent area for future studies. Individual cognitive interviews and focus group interviews could be applied in future studies to the Spanish-translated food insecurity scale and the adolescent food insecurity scale. Relatedly, future research on household food insecurity discordance should expand its measurements to include both parents (i.e., mother and father), more than one focal child in the household, and older members of the family (i.e., grandparents). Specifically, given that mothers and fathers interpret the food security items differently [86], adding assessments of household food security at the family level may explain why mothers report higher food insecurity than fathers [10, 87, 88]. Additionally, a longitudinal follow-up study would be beneficial in exploring whether the parent–adolescent household food insecurity discordance tends to improve or become worse over time. Furthermore, qualitative studies (i.e., case studies, focus groups) could also contribute to a better understanding of the sociocultural underpinnings—unique contexts that cannot be measured in the USDA household food security modules—and cultural values that may be influencing parents or adolescents' behavior (i.e., food reduction, discrete food management, and food insecurity secrecy) and reports of food discordance in the household.

### 5.6. Implications for Public Health Nursing

This study demonstrates that attestations of food security status cannot necessarily be taken at face value by nurses. While the study cannot speak for all populations, among Latinos, food insecurity discordance was observed in nearly one-third of the interviewed dyads. To the extent that food security effects assessments of nutrition levels, healthy diet, and overall health for minors, it is advisable for nurses to rely on input from both the minor and their parent or guardian (although the minor should be asked separately if at all possible). In addition, this study highlights the importance of considering hunger and food insecurity in managing antisocial and problematic behavior and that behavioral responses to food insecurity may differ by gender. In the present study, significantly higher antisocial attitudes were found among food-insecure girls, whether or not the parent was aware, while higher antisocial behavior was seen among boys only when they seemed secure in their food situation but the parent was not. If food insecurity or discordance is discovered, either through direct questioning or suspected due to behavioral issues, we would recommend that nurses have names and locations of local food banks and distribution centers handy, as well as advice for adolescents to discretely present the information to their parents in the case of adolescent food discordance.

## 6. Conclusion

The present research provides insight into household food insecurity concordance and discordance as a family experience among Latino families. Specifically, our study highlights the negative implications of household food insecurity discordance on adolescent girls' and adolescent boys' antisocial behavior. Findings show that parents can not only be unaware of the food insecurity experiences of their adolescents, but that this ignorance negatively affects Latino adolescent girls' antisocial behavior. Additionally, Latino girls' antisocial behavior is worsened in cases of food insecurity concordance, whereas boys only exhibit greater antisocial behavior when they report food security but their parents do not. Cultural circumstances specific to Latino populations may drive these dynamics. Nurses should take the possibility of food insecurity discordance and gender-specific reactions to food security into consideration when assessing and treating the physical and mental health of Latino adolescent patients.

## Figures and Tables

**Table 1:** Descriptive statistics of parent–adolescent dyads (*N* = 69 dyads; 138 total participants).

Characteristics	Parent (*N* = 69)		Adolescent (*N* = 69)	
	*n* or *M*, (SD), [Min, Max]	%	*n* or *M*, (SD), [Min, Max]	%
Food secure	50	72.5	53	76.8
Food insecure	19	27.5	16	23.2
Adolescent antisocial behavior			1.38 (0.35) [1–2.5]	
Age, mean (SD) [range]	38.46 (6.49) [26–65]		14.26 (2.01) [11–19]	
Gender				
Girl	62	89.9	30	43.5
Boy	7	10.1	39	56.5
Educational level (years)				
5–6			10	1.5
7–9			31	44.9
≥ 10			28	40.6
No high school graduation	46	66.7		
Graduation from high school	23	33.3		
Marital status				
Married/living as married	61	88		
Unmarried	8	12		
Relation to focal adolescent				
Biological mother	55	79.7		
Other	11	15.9		
N/A	3	4.3		
Working more than one job				
Yes	22	32		
No	47	68		
Household income per year				
Less than $29,999	46	66.7		
$30,000–$99,999	23	33.3		

**Table 2:** Adjusted means for antisocial behavior by gender and food security status.

Adolescent Gender	Adolescent food insecurity discordance		Parental food insecurity discordance		Household food security concordance		Household food insecurity concordance		Overall	
	*M*	*n*	*M*	*n*	*M*	*n*	*M*	*n*	*M*	*n*
Girl	1.65^a^	4	1.19^1^	3	1.21^b^	19	1.58^a^	4	1.41	30
Boy	1.25^a^	5	1.70^b2^	9	1.34^a^	22	1.63	3	1.48	39
Overall	1.45	9	1.45	12	1.28^a^	41	1.61^b^	7	1.44	69

*Note*: Within each row, means with a different letter in their superscript are significantly different from one another. Within each column, means with a different number in their superscript are significantly different from one another. Alpha set at 0.05 for all tests, with specific *p* values reported in text.

## Data Availability

The data that support the findings of this study are available from the corresponding author upon reasonable request.

## Endnotes

^1^We use the term “Latino” throughout this manuscript rather than “Latinx” or “Latino/a” because of several public comments by the Latin American community rejecting these phrases [89] on the grounds that gender is built into the vocabulary and basic grammar of the spoken language, retrieved from https://www.usatoday.com/story/news/nation/2019/06/29/latina-latino-latinx-hispanic-what-do-they-mean/1596501001/.
